# Thoracoscopy for trauma

**DOI:** 10.4103/0970-2113.80313

**Published:** 2011

**Authors:** Laleng M. Darlong

**Affiliations:** *Department of Thoracic Surgery and Thoracic Surgical Oncology, Fortis Hospital, Noida, NCR, India. E-mail: lmdarlong@gmail.com*

Thoracic injuries following trauma is an important cause of morbidity and mortality.[1] There is no doubt that urgent thoracotomy remains the access of choice in patients with shock due to life-threatening thoracic injuries. However, a majority of hemodynamically stable thoracic injuries can be managed with tube thoracostomy initially or as a definitive care.[2] It is in this group of hemodynamically stable patients that thoracoscopy or video-assisted thoracic surgery (VATS) has a definite role for timely assessment and treatment of intrathoracic injuries. As a minimal access procedure, it reduces the morbidity associated with a negative or non-therapeutic thoracotomy and also avoids chronic post-thoracotomy pain observed in 5 to 25% of patients.[3]

An improvement in video imaging and increasing expertise in thoracoscopy for routine thoracic surgical procedure has been observed in the last decade, which has increased its application in the setting of chest injuries among thoracic surgeons and trauma surgeons.[4] Before operation, all patients must undergo a complete imaging evaluation, including plain chest roentgenogram, a computed tomography (CT) angiography scan of the chest, and abdominal assessment with ultrasound imaging, CT scan, or both.

The procedure is performed under general anesthesia with single lung ventilation using double-lumen tube. It can be truly an endoscopic procedure using three incisions 1 to 2 cm long at the intercostals spaces known as thoracoscopy. When a 5-cm utility incision without rib spreading is used at the fourth or fifth anterolateral chest wall, it is known as video assisted thoracic surgery (VATS).

A conversion to open thoracotomy should be promptly done should the situation demand and not to be regarded as a failure. More complex cases are best managed by a team comprising of thoracic surgeons with support of heart lung machine by cardiac surgeons, respiratory physicians, physiotherapists and dedicated nurses for the best postoperative outcome.

Thoracoscopy or VATS is ideal for evacuation of residual hemothorax following tube thoracostomy, managing thoracic bleeding in stable patients, prevention and treatment of empyema, assessment and repair of persistent air leak, diagnosis and repair of diaphragmatic injuries and also removal of foreign bodies such as glass or metal fragments.[5,6]

Following chest trauma, retained hemothorax which can occur in up to 18% of patients initially treated with tube thoracostomy is among the commonest complications and can significantly increase the morbidity and hospital costs.[7] Several authors have cited 500 ml of retained blood clot as a threshold for operative intervention which should be done as early as possible from the 2^nd^ to 10^th^ day of tube drainage.[8,9] This avoids complication such as fibrothorax, lung entrapment or empyema arising due to undrained intrapleural clot.

In patients who are hemodynamically stable but continue to bleed from the thoracostomy tube, a carefully performed thoracoscopy, gives valuable information about parietal or pulmonary bleeding and managed accordingly.

A persistent air-leak or bronchopleural fistula following trauma should be managed early using thoracoscopy, before pleural adhesions sets in limiting utility of thoracoscopy.[10]

In suspected injuries of the mediastinum with the availability of high-resolution CT scan, thoracoscopy is infrequently used for a diagnosis. However, when such facility is not available, thoracoscopy can be both diagnostic and therapeutic to visualize the integrity of the trachea, esophagus and pericardium.[11]

For suspected diaphragmatic injuries, thoracoscopy is both diagnostic and therapeutic by visualizing the diaphragm including the posterior recesses, an area not often seen well with the laparoscope.[12]

Thoracoscopy can also be used for retrieval of foreign bodies within the thorax, such as glass fragments avoiding a thoracotomy.[13] This issue of Lung India contains an article on retrieval of foreign body using VATS.[14]

In the modern era of trauma management, it can be concluded that thoracoscopy for chest trauma should be considered an essential tool and not a luxury for thoracic or trauma surgeons.
